# An Increasing Danger of Zoonotic Orthopoxvirus Infections

**DOI:** 10.1371/journal.ppat.1003756

**Published:** 2013-12-05

**Authors:** Sergei N. Shchelkunov

**Affiliations:** 1 State Research Center of Virology and Biotechnology VECTOR, Koltsovo, Novosibirsk Oblast, Russia; 2 Novosibirsk State University, Novosibirsk, Russia; The Fox Chase Cancer Center, United States of America

## Abstract

On May 8, 1980, the World Health Assembly at its 33^rd^ session solemnly declared that the world and all its peoples had won freedom from smallpox and recommended ceasing the vaccination of the population against smallpox. Currently, a larger part of the world population has no immunity not only against smallpox but also against other zoonotic orthopoxvirus infections. Recently, recorded outbreaks of orthopoxvirus diseases not only of domestic animals but also of humans have become more frequent. All this indicates a new situation in the ecology and evolution of zoonotic orthopoxviruses. Analysis of state-of-the-art data on the phylogenetic relationships, ecology, and host range of orthopoxviruses—etiological agents of smallpox (variola virus, VARV), monkeypox (MPXV), cowpox (CPXV), vaccinia (VACV), and camelpox (CMLV)—as well as the patterns of their evolution suggests that a VARV-like virus could emerge in the course of natural evolution of modern zoonotic orthopoxviruses. Thus, there is an insistent need for organization of the international control over the outbreaks of zoonotic orthopoxvirus infections in various countries to provide a rapid response and prevent them from developing into epidemics.

The genus *Orthopoxvirus* of the family *Poxviridae* comprises the species variola (smallpox) virus (VARV), with human as its only sensitive host; zoonotic species monkeypox virus (MPXV), cowpox virus (CPXV), vaccinia virus (VACV), and camelpox virus (CMLV); and several others. These orthopoxviruses are immunologically cross-reactive and cross-protective, so that infection with any member of this genus provides protection against infection with any other member of the genus [1], [2]. Traditionally, the species of the *Orthopoxvirus* genus have been named primarily according to the host animal from which they were isolated and identified based on a range of biological characteristics [1]. Most frequently, zoonotic orthopoxviruses have been initially isolated from animals immediately close to humans being incidental hosts for the virus, the natural carriers of which are, as a rule, wild animals. Correspondingly, the name of an orthopoxvirus species does not reflect the actual animal that is its natural reservoir.

With accumulation of the data on complete genome nucleotide sequences for various strains of orthopoxvirus species, it has been found that an interesting feature of the orthopoxvirus genomes is the presence of genes that are intact in one species but fragmented or deleted in another [3]–[8]. These data confirm the concept of a reductive evolution of orthopoxviruses, according to which the gene loss plays an important role in the evolutionary adaptation of progenitor virus to a particular environmental niche (host) and emergence of new virus species [9]. CPXV has the largest genome of all the modern representatives of the genus *Orthopoxvirus*, and this genome contains all the genes found in the other species of this genus [2], [4], [10]–[12]. Therefore, *Cowpox virus* was proposed as the closest of all the modern species to the progenitor virus for the genus *Orthopoxvirus*, while the remaining species, *Variola virus* included, had appeared as a result of multistage reductive evolution [4], [9], [13].

VARV, the most pathogenic species for humans, has the smallest genome of all the orthopoxviruses [2]–[7]. This suggests a potential possibility for emergence of a VARV-like variant from the currently existing zoonotic orthopoxviruses with longer genomes in the course of natural evolution. It is known that although mutational changes are rather a rare event for the poxvirus DNA [13], characteristic of these viruses is the possibility of intermolecular and intramolecular recombinations, as well as genomic insertions and deletions [14], [15]. It has been recently found that duplication/amplification of genomic segments is typical of poxviruses, and in the case of a certain selective pressure (for example, host antiviral defenses), certain genes are able to relatively rapidly accumulate mutations that would provide the virus adaptation to new conditions, including a new host [16].

The conducted analysis of the available archive data on smallpox and the history of ancient civilizations as well as the newest data on the evolutionary relationships of orthopoxviruses has allowed me to suggest the hypothesis that smallpox could have repeatedly emerged in the past via evolutionary changes of a zoonotic progenitor virus [17].

Because of the cessation of the vaccination against smallpox after its eradication 35 years ago, a tremendous part of the world human population currently has no immunity not only against smallpox, but also against any other zoonotic orthopoxvirus infections. This new situation allows orthopoxviruses to circulate in the human population and, as a consequence, should alter several established concepts on the ecology and range of sensitive hosts for various orthopoxvirus species.

The most intricate case is the origin of VACV. For many decades, VACV has been used for vaccinating humans against smallpox, and it was considered that this virus, *variolae vaccinae*, originates from zoonotic CPXV, introduced to immunization practice by Jenner as early as 1796 [1]. Only in the 20^th^ century was it found out that the orthopoxvirus strains used for smallpox vaccination significantly differ in their properties from both the natural CPXV isolates recovered from cows and the other orthopoxvirus species examined by that time [18]. Correspondingly, they were regarded as a separate species, *Vaccinia virus* [19]. Moreover, it was inferred that the VACV natural reservoir was unknown and numerous hypotheses attempted to explain the origin of this virus while passaging progenitor viruses in animals in the process of vaccine production [1], [2], [20].

The issue of VACV origin was somewhat clarified after sequencing the complete genome of horsepox virus (HSPV) [21], which appeared to be closely related to the sequenced VACV strains. Only after this was attention paid to the fact that Jenner specified the origin of his vaccine from an infection of the heels of horses (“grease”) and indicated that the vaccine became more suitable for human use after passage through the cow [20]. This suggests that VACV may originate from a zoonotic HSPV, which naturally persisted concurrently with CPXV. Some facts suggest that the infectious materials not only from cow lesions but also from horse lesions were used for smallpox vaccination in the 19^th^ century. The vaccine lymph from the horse gave the most satisfactory results in inducing an anti-smallpox immunity as well as less side reactions [1]. By all accounts, they gradually commenced using HSPV isolates for smallpox vaccination, the future generations of which recovered decades later were ascribed to the separate species *Vaccinia virus* [19], rather than CPXV for smallpox vaccination everywhere.

Since the 1960s, VACVs have been repeatedly isolated in Brazil [22]. The first VACV isolates were recovered from wild rodents (sentinel mice and rice rat) [23]. Since 1999, an ever-increasing number of exanthematous outbreaks affecting dairy cows and their handlers have been recorded [24]–[27], supplemented recently with outbreaks among horses [28], [29]. Several VACV strains have been isolated during these outbreaks from cows, horses, humans, and rodents [22], [27], [28], [30], [31]. The questions that arise are when and how VACV entered Brazil and the wild nature of the American continent. The more widespread point of view is that VACV strains could be transmitted from vaccinated humans to domestic animals and further to wild ones with subsequent adaptation to the rural environment [22]. My standpoint implies that HSPV/VACV could have been repeatedly accidentally imported from Europe to South America with the infected horses or rodents to be further introduced into wildlife. Possibly, the latter hypothesis more adequately reflects the actual pathway of VACV transmission to the Brazilian environment, since recent phylogenetic studies have suggested an independent origin for South American VACV isolates, distinct from the vaccine strains used on this continent during the WHO smallpox eradication campaign [22], [32]. Presumably, genome-wide sequencing of the viruses will give a more precise answer to the origin of VACV variants isolated in Brazil.

In the past, the outbreaks of buffalopox had occurred frequently in various states of India as well as in Pakistan, Bangladesh, Indonesia, Egypt, and other countries [33]. The causative agent, buffalopox virus (BPXV), is closely related to VACV and affiliated with the species *Vaccinia virus*, genus *Orthopoxvirus* [2], [34]. Recently, mass outbreaks of buffalopox in domestic buffaloes along with severe zoonotic infection in milk attendants were recorded at various places in India [35], [36]. In several buffalopox outbreaks, the BPXV-caused infections were recorded in cows in the same herds [37]. An increase in BPXV transmission to different species, including buffaloes, cows, and humans, suggests the reemergence of zoonotic buffalopox infection [35], [38]. The buffalopox outbreaks recorded in different distant regions of India are likely to suggest the presence of an abundant natural BPXV reservoir represented by wild animals, most probably rodents. Correspondingly, it is of the paramount importance to perform a large-scale study of the presence of orthopoxviruses in wild animals of India.

Thus, yet incomplete data on the modern ecology of VACV and BPXV allow for speculation that the orthopoxviruses belonging to the species *Vaccinia virus* have a wide host range, are zoonotic, are currently spread over large areas in Eurasia and South America, and that their natural carriers are several rodents.

CPXV has relatively low pathogenicity for humans but has a wide range of sensitive animal hosts [2], [39]. Human cowpox is a rare sporadic disease, which develops when CPXV is transmitted from an infected animal to human [2], [40]. This disease is mainly recorded in Europe. In wildlife, CPXV carriers are asymptomatically infected rodents [41], [42]. During the last two decades, reports on an increasing number of CPXV infections in cats, rats, exotic animals, and humans have been published [43]–[47]. Comparative studies of the properties of CPXV isolates recovered from various hosts at different times and in several geographic zones have shown sufficient intraspecific variations [2], [48], [49]. A recent phylogenetic analysis of the complete genomes of 12 CPXV strains recovered from humans and several animal species suggests that they be split into two major *Cowpox virus*–like and *Vaccinia virus*–like clades [50]. This means that the criteria of the separation of orthopoxviruses into these two species should be corrected.

MPXV is a zoonotic virus causing a human infection similar to smallpox in its clinical manifestations with a lethality rate of 1–8% [51]. The natural reservoir of MPXV is various species of African rodents [8], [10]. The active surveillance data in the same health zone (Democratic Republic of Congo) from the 1980s to 2006–2007 suggest a 20-fold increase in human monkeypox incidence 30 years after the cessation of the smallpox vaccination campaign [52]. This poses the question of whether MPXV can acquire the possibility of a high human-to-human transmission rate, characteristic of VARV, under conditions of a long-term absence of vaccination and considerably higher incidence of human infection. If this occurs, humankind will face a problem considerably more complex than with the smallpox eradication. First and foremost, this is determined by the fact that MPXV, unlike VARV, has its natural reservoir represented by numerous African rodents [2], [53].

In its biological properties and according to the data of phylogenetic analysis of the complete virus genomic sequence, CMLV is closest to VARV, the causative agent of smallpox, as compared with the other orthopoxvirus species [1], [8]. Camelpox is recognized as one of the most important viral diseases in camels. This infection was first described in India in 1909. Subsequently, camelpox outbreaks have been reported in many countries of the Middle East, Asia, and Africa [54], [55]. Until recently, it has been commonly accepted that the host range of CMLV is confined to one animal species, camels [1], [55]. However, the first human cases of camelpox have been recently confirmed in India [56]. This suggests that camelpox could be a zoonotic disease. Since camelpox outbreaks occur irregularly in distant regions of the world and the viruses isolated during these outbreaks display different degrees of virulence [55], it is possible to postulate the presence of a wildlife animal reservoir of CMLV other than camels. Since the camelpox outbreaks are usually associated with the rainy season of the year, when rodents are actively reproducing, it is likely that rodents could be the natural carriers of CMLV.

It is known that most of the emerging human pathogens originate from zoonotic pathogens [57]–[59]. Many viruses do not cause the disease in their natural reservoir hosts but can be highly pathogenic when transmitted to a new host species. Emerging and reemerging human pathogens more often are those with broad host ranges. The viruses able to infect many animal species are evolutionarily adapted to utilizing different cell mechanisms for their reproduction and, thus, can extend/change their host range with a higher probability [58].

There are no fundamental prohibitions for the possible reemergence of smallpox or a similar human disease in the future as a result of natural evolution of the currently existing zoonotic orthopoxviruses. An ever-increasing sensitivity of the human population to zoonotic orthopoxviruses, resulting from cessation of the mass smallpox vaccination, elevates the probability for new variants of these viruses, potentially dangerous for humans, to emerge. However, the current situation is radically different from the ancient one, since many outbreaks of orthopoxvirus infections among domestic animals and humans are recorded and studied.

Recently, the efforts of scientists under WHO control are directed to the development of state-of-the-art methods for VARV rapid identification as well as design of new generation safe smallpox vaccines and drugs against VARV and other orthopoxviruses [60]. The designed promising anti-orthopoxvirus drugs display no pronounced virus species specificity. Therefore, they are applicable in the outbreaks caused by any orthopoxvirus species. International acceptance of the designed highly efficient anti-orthopoxvirus drugs ST-246 and CMX001 [60] is of paramount importance.

In the areas of high incidence of zoonotic orthopoxviral infections, it would be purposeful to vaccinate domestic and zoo animals as well as the persons closely associated with them using state-of-the-art safe vaccines based on VACV, which has a wide range of sensitive hosts. This would considerably decrease the likelihood for such infections to spread from wildlife into the human environment.

In the African region endemic for monkeypox, which also displays a high rate of HIV infection, the population could be vaccinated with the VACV strain MVA, which has been recently demonstrated to be safe even for HIV-infected persons [61].

Taking into account the above mentioned increased incidence of outbreaks of animal and human orthopoxvirus infections and their potential danger, it is important to accelerate organization of the international Smallpox Laboratory Network, discussed by the WHO Advisory Committee on Variola Virus Research [62], [63], and orient this network to express diagnosing not only of VARV but also of other zoonotic orthopoxviruses. This will provide constant monitoring of these infections in all parts of the world and make it possible to prevent the development of small outbreaks into expanded epidemics, thereby decreasing the risk of evolutional changes and emergence of an orthopoxvirus highly pathogenic for humans.

The international system for clinical sampling and identification of infectious agents has been worked out and optimized while implementing the global smallpox eradication program under the aegis of the WHO as well as anti-epidemic measures and methods for mass vaccination [1]. The accumulated experience is of paramount importance for the establishment of international control not only over currently existing orthopoxvirus infections but also other emerging and reemerging diseases.

## References

[1] Fenner F, Henderson DA, Arita I, Jezek Z, Ladnyi ID (1988) Smallpox and its eradication. Geneva: World Health Organization. 1460 p.

[2] Shchelkunov SN, Marennikova SS, Moyer RW (2005) Orthopoxviruses pathogenic for humans. New York: Springer. 425 p.

[3] Shchelkunov SN, Resenchuk SM, Totmenin AV, Blinov VM, Marennikova SS, et al (1993) Comparison of the genetic maps of variola and vaccinia viruses. FEBS Lett 327: 321–324. 8394246 10.1016/0014-5793(93)81013-p

[4] Shchelkunov SN, Safronov PF, Totmenin AV, Petrov NA, Ryazankina OI, et al (1998) The genomic sequence analysis of the left and fight species-specific terminal region of a cowpox virus strain reveals unique sequences and a cluster of intact ORFs for immunomodulatory and host range proteins. Virology 243: 432–460. 9568042 10.1006/viro.1998.9039

[5] Shchelkunov SN, Totmenin AV, Loparev VN, Safronov PF, Gutorov VV, et al (2000) Alastrim smallpox variola minor virus genome DNA sequences. Virology 266: 361–386. 10639322 10.1006/viro.1999.0086

[6] Shchelkunov SN, Totmenin AV, Babkin IV, Safronov PF, Ryazankina OI, et al (2001) Human monkeypox and smallpox viruses: genomic comparison. FEBS Lett 509: 66–70. 11734207 10.1016/S0014-5793(01)03144-1 PMC9533818

[7] Shchelkunov SN, Totmenin AV, Safronov PF, Mikheev MV, Gutorov VV, et al (2002) Analysis of the monkeypox virus genome. Virology 297: 172–194. 12083817 10.1006/viro.2002.1446 PMC9534300

[8] Gubser C, Smith GL (2002) The sequence of camelpox virus shows it is most closely related to variola virus, the cause of smallpox. J Gen Virol 83: 855–872. 11907336 10.1099/0022-1317-83-4-855

[9] Hendrickson RC, Wang C, Hatcher EL, Lefkowitz EJ (2010) Orthopoxvirus genome evolution: the role of gene loss. Viruses 2: 1933–1967. 21994715 10.3390/v2091933 PMC3185746

[10] Meyer H, Totmenin A, Gavrilova E, Shchelkunov S (2005) Variola and camelpox virus-specific sequences are part of a single large open reading frame identified in two German cowpox virus strains. Virus Res 108: 39–43. 15681053 10.1016/j.virusres.2004.07.011

[11] Gubser C, Hue S, Kellam P, Smith GL (2004) Poxvirus genomes: a phylogenetic analysis. J Gen Virol 85: 105–117. 14718625 10.1099/vir.0.19565-0

[12] Shchelkunov SN (2012) Orthopoxvirus genes that mediate disease virulence and host tropism. Adv Virol 2012: 524743. 22899927 10.1155/2012/524743 PMC3413996

[13] Shchelkunov SN (2009) How long ago did smallpox virus emerge? Arch Virol 154: 1865–1871. 19882103 10.1007/s00705-009-0536-0

[14] Shchelkunov SN, Totmenin AV (1995) Two types of deletions in orthopoxvirus genomes. Virus Genes 9: 231–245. 7597802 10.1007/BF01702879

[15] Coulson D, Upton C (2011) Characterization of indels in poxvirus genomes. Virus Genes 42: 171–177. 21153876 10.1007/s11262-010-0560-x

[16] Elde NC, Child SJ, Eickbush MT, Kitzman JO, Rogers KS, et al (2012) Poxviruses deploy genomic accordions to adapt rapidly against host antiviral defenses. Cell 150: 831–841. 22901812 10.1016/j.cell.2012.05.049 PMC3499626

[17] Shchelkunov SN (2011) Emergence and reemergence of smallpox: the need in development of a new generation smallpox vaccine. Vaccine 29S: D49–53. 10.1016/j.vaccine.2011.05.037 22185833

[18] Downie AW (1939) The immunological relationship of the virus of spontaneous cowpox to vaccinia virus. Br J Exp Pathol 20: 158–176.

[19] Downie AW, Dumbell KR (1956) Pox viruses. Annu Rev Microbiol 10: 237–252. 13363362 10.1146/annurev.mi.10.100156.001321

[20] Baxby D (1977) The origins of vaccinia virus. J Infect Dis 136: 453–455. 198484 10.1093/infdis/136.3.453

[21] Tulman ER, Delhon G, Afonso CL, Lu Z, Zsak L, et al (2006) Genome of horsepox virus. J Virol 80: 9244–9258. 16940536 10.1128/JVI.00945-06 PMC1563943

[22] Trindade GS, Emerson GL, Carroll DS, Kroon EG, Damon IK (2007) Brazilian vaccinia viruses and their origins. Emerg Infect Dis 13: 965–972. 18214166 10.3201/eid1307.061404 PMC2878226

[23] da Fonseca FG, Trindade GS, Silva RL, Bonjardim CA, Ferreira PC, et al (2002) Characterization of a vaccinia-like virus isolated in a Brazilian forest. J Gen Virol 83: 223–228. 11752719 10.1099/0022-1317-83-1-223

[24] Damaso CR, Esposito JJ, Condit RC, Moussatche N (2000) An emergent poxvirus from humans and cattle in Rio de Janeiro State: Cantagalo virus may derive from Brazilian smallpox vaccine. Virology 277: 439–449. 11080491 10.1006/viro.2000.0603

[25] Leite JA, Drumond BP, Trindade GS, Lobato ZI, da Fonseca FG, et al (2005) Passatempo virus, a vaccinia virus strain, Brazil. Emerg Infect Dis 11: 1935–1938. 16485483 10.3201/eid1112.050773 PMC3367646

[26] Trindade GS, Lobato ZI, Drumond BP, Leite JA, Trigueiro RC, et al (2006) Isolation of two vaccinia virus strains from a single bovine vaccinia outbreak in rural area from Brazil: implications on the emergence of zoonotic orthopoxviruses. Am J Trop Med Hyg 75: 486–490. 16968926

[27] Megid J, Borges IA, Abrahão JS, Trindade GS, Appolinario CM, et al (2012) Vaccinia virus zoonotic infection, Sao Paulo State, Brazil. Emerg Infect Dis 18: 189–191. 22260819 10.3201/eid1801.110692 PMC3310104

[28] Campos RK, Brum MC, Nogueira CE, Drumond BP, Alves PA, et al (2011) Assessing the variability of Brazilian Vaccinia virus isolates from a horse exanthematic lesion: coinfection with distinct viruses. Arch Virol 156: 275–283. 21080203 10.1007/s00705-010-0857-z

[29] Cargnelutti JF, Schmidt C, Masuda EK, Nogueira PR, Weiblen R, et al (2012) Vaccinia viruses isolated from skin infection in horses produced cutaneous and systemic disease in experimentally infected rabbits. Res Vet Sci 93: 1070–1075. 22244689 10.1016/j.rvsc.2011.12.016

[30] Abrahão JS, Guedes MIM, Trindade GS, Fonseca FG, Campos RK, et al (2009) One more piece in the VACV ecological puzzle: could peridomestic rodents be the link between wildlife and bovine vaccinia outbreaks in Brazil? PLoS ONE 4: e7428 doi:10.1371/journal.pone.0007428. 19838293 10.1371/journal.pone.0007428 PMC2758550

[31] D'Anunciação L, Guedes MIM, Oliveira TL, Rehfeld I, Bonjardim CA, et al (2012) Filling one more gap: experimental evidence of horizontal transmission of Vaccinia virus between bovines and rodents. Vector Borne Zoonotic Dis 12: 61–64. 21923268 10.1089/vbz.2011.0671

[32] Drumond BP, Leite JA, da Fonseca FG, Bonjardim CA, Ferreira PC, et al (2008) Brazilian Vaccinia virus strains are genetically divergent and differ from the Lister vaccine strain. Microbes Infect 10: 185–197. 18248758 10.1016/j.micinf.2007.11.005

[33] Lal SM, Singh IP (1977) Buffalopox - a review. Trop Anim Health Prod 9: 107–112. 198924 10.1007/BF02236389

[34] Singh RK, Hosamani M, Balamurugan V, Satheesh CC, Rasool TJ, et al (2006) Comparative sequence analysis of envelope protein genes of Indian buffalopox virus isolates. Arch Virol 151: 1995–2005. 16625321 10.1007/s00705-006-0761-8

[35] Bhanuprakash V, Venkatesan G, Balamurugan V, Hosamani M, Yogisharadhya R, et al (2010) Zoonotic infections of buffalopox in India. Zoonoses Public Health 57: e149–155. 20042058 10.1111/j.1863-2378.2009.01314.x

[36] Venkatesan G, Balamurugan V, Prabhu M, Yogisharadhya R, Bora DP, et al (2010) Emerging and re-emerging zoonotic buffalopox infection: a severe outbreak in Kolhapur (Maharashtra), India. Vet Ital 46: 439–448. 21120799

[37] Yadav S, Hosamani M, Balamurugan V, Bhanuprakash V, Singh RK (2010) Partial genetic characterization of viruses isolated from pox-like infection in cattle and buffaloes: evidence of buffalo pox virus circulation in Indian cows. Arch Virol 155: 255–261. 19956986 10.1007/s00705-009-0562-y

[38] Bera BCh, Shanmugasundaram K, Barua S, Anand T, Riyesh T, et al (2012) Sequence and phylogenetic analysis of host-range (E3L, K3L, and C7L) and structural protein (B5R) genes of buffalopox virus isolates from buffalo, cattle, and human in India. Virus Genes 45: 488–498. 22872567 10.1007/s11262-012-0788-8

[39] Baxby D (1977) Poxvirus hosts and reservoirs. Arch Virol 55: 169–179. 202224 10.1007/BF01319903

[40] Essbauer S, Pfeffer M, Meyer H (2010) Zoonotic poxviruses. Vet Microbiol 140: 229–236. 19828265 10.1016/j.vetmic.2009.08.026 PMC9628791

[41] Kinnunen PM, Henttonen H, Hoffmann B, Kallio ER, Korthase C, et al (2011) Orthopox virus infections in Eurasian rodents. Vector Borne Zoonot Dis 11: 1133–1140. 10.1089/vbz.2010.0170 21453121

[42] Tryland M, Okeke MI, Segerstad CH, Morner T, Traavik T, et al (2011) Orthopoxvirus DNA in Eurasian lynx, Sweden. Emerg Infect Dis 17: 626–632. 21470451 10.3201/eid1704.091899 PMC3377389

[43] Campe H, Zimmermann P, Glos K, Bayer M, Bergemann H, et al (2009) Cowpox virus transmission from pet rats to humans, Germany. Emerg Infect Dis 15: 777–780. 19402967 10.3201/eid1505.090159 PMC2687013

[44] Ninove L, Domart Y, Vervel C, Voinot C, Salez N, et al (2009) Cowpox virus transmission from pet rats to humans, France. Emerg Infect Dis 15: 781–784. 19402968 10.3201/eid1505.090235 PMC2686997

[45] Kurth A, Straube M, Kuczka A, Dunsche AJ, Meyer H, et al (2009) Cowpox virus outbreak in banded mongooses (*Mungos mungo*) and jaguarundis (*Herpailurus yagouaroundi*) with a time-delayed infection to humans. PLoS ONE 4: e6883 doi:10.1371/journal.pone.0006883. 19727399 10.1371/journal.pone.0006883 PMC2731879

[46] Cardeti G, Brozzi A, Eleni C, Polici N, D'Alterio G, et al (2011) Cowpox virus in llama, Italy. Emerg Infect Dis 17: 1513–1515. 21801638 10.3201/eid1708.101912 PMC3381539

[47] Favier AL, Flusin O, Lepreux S, Fleury H, Labreze C, et al (2011) Necrotic ulcerated lesion in a young boy caused by cowpox virus infection. Case Rep Dermatol 3: 186–194. 22110431 10.1159/000331426 PMC3219450

[48] Pilaski J, Rosen A, Darai G (1986) Comparative analysis of the genomes of orthopoxviruses isolated from elephant, rhinoceros, and okapi by restriction enzymes. Arch Virol 88: 135–142. 3954598 10.1007/BF01310898

[49] Kaysser P, von Bomhard W, Dobrzykowski L, Meyer H (2010) Genetic diversity of feline cowpox virus, Germany 2000–2008. Vet Microbiol 141: 282–288. 19879071 10.1016/j.vetmic.2009.09.029

[50] Carroll DS, Emerson GL, Li Y, Sammons S, Olson V, et al (2011) Chasing Jenner's vaccine: revisiting *Cowpox virus* classification. PLoS ONE 6: e23086 doi:10.1371/journal.pone.0023086. 21858000 10.1371/journal.pone.0023086 PMC3152555

[51] Jezek Z, Fenner F (1988) Human monkeypox. Monog Virol 17: 1–140.

[52] Rimoin AW, Mulembakani PM, Jonston SC, Lloyd Smith JO, Kisalu NK, et al (2010) Major increase in human monkeypox incidence 30 years after smallpox vaccination campaigns cease in the Democratic Republic of Congo. Proc Natl Acad Sci U S A 107: 16262–16267. 20805472 10.1073/pnas.1005769107 PMC2941342

[53] Di Giulio DB, Eckburg PB (2004) Human monkeypox: an emerging zoonosis. Lancet Infect Dis 4: 15–25. 14720564 10.1016/S1473-3099(03)00856-9 PMC9628772

[54] Al-Zi'Abi O, Hishikawa H, Meyer H (2007) The first outbreak of camelpox in Syria. J Vet Med Sci 69: 541–543. 17551230 10.1292/jvms.69.541

[55] Duraffour S, Meyer H, Andrei G, Snoeck R (2011) Camelpox virus. Antiviral Res 92: 167–186. 21945248 10.1016/j.antiviral.2011.09.003

[56] Bera BC, Shanmugasundaram K, Barua S, Venkatesan G, Virmani N, et al (2011) Zoonotic cases of camelpox infection in India. Vet Microbiol 152: 29–38. 21571451 10.1016/j.vetmic.2011.04.010

[57] Domingo E (2010) Mechanisms of viral emergence. Vet Res 41: 38. 20167200 10.1051/vetres/2010010 PMC2831534

[58] Parrish CR, Holmes EC, Morens DM, Park EC, Burke DS, et al (2008) Cross-species virus transmission and the emergence of new epidemic diseases. Microbiol Mol Biol Rev 72: 457–470. 18772285 10.1128/MMBR.00004-08 PMC2546865

[59] Wolfe ND, Dunavan CP, Diamond J (2007) Origins of major human infectious diseases. Nature 447: 279–283. 17507975 10.1038/nature05775 PMC7095142

[60] Alcami A, Damon I, Evans D, Huggins JW, Hughes C, et al. (2010) Scientific review of variola virus research, 1999–2010. Geneva: World Health Organization. WHO/HSE/GAR/BDP/2010.3.

[61] Greenberg RN, Overton ET, Haas DW, Frank I, Goldman M, et al (2013) Safety, immunogenicity, and surrogate markers of clinical efficacy for modified vaccinia Ankara as a smallpox vaccine in HIV-infected subjects. J Infect Dis 207: 749–758. 23225902 10.1093/infdis/jis753 PMC3611764

[62] WHO Advisory Committee on Variola Virus Research (2011) Report of the thirteenth meeting, Geneva, Switzerland, 31 October–1 November 2011. Geneva: World Health Organization. WHO/HSE/GAR/BDP/2011.2.

[63] WHO Advisory Committee on Variola Virus Research (May 2013) Report of the fourteenth meeting, Geneva, Switzerland, 16–17 October 2012. Geneva: World Health Organization. WHO/HSE/PED/CED/2013.1.

